# Chronic Kidney Disease and Associated Factors among Diabetic Patients at the Diabetic Clinic in a Police Hospital, Addis Ababa

**DOI:** 10.4314/ejhs.v32i2.11

**Published:** 2022-03

**Authors:** Muna Abdulkadr, Hailu Merga, Biru Abdissa Mizana, Gemechu Terefe, Lamessa Dube

**Affiliations:** 1 Nephrologist, Ethiopian Police Hospital, Addis Ababa, Ethiopia; 2 Department of Epidemiology, Institute of Health, Jimma University, Jimma, Ethiopia; 3 School of Midwifery, Institute of Health, Jimma University, Jimma, Ethiopia

**Keywords:** Chronic kidney disease, Police Hospital, Addis Ababa

## Abstract

**Background:**

Diabetes mellitus remains the leading cause of end-stage renal disease in most countries in the world. In Ethiopia, renal complications of diabetes may remain unrecognized due to limited diagnostic resources. As a result, the prevalence of chronic kidney disease among adult diabetics in Ethiopia has not been well described. Hence, this study was aimed at assessing the prevalence of chronic kidney disease and associated factors among diabetic patients who attended the federal police hospital diabetic clinic in Addis Ababa

**Methods:**

A cross-sectional study was conducted among 362 Diabetes Mellitus. Data were collected using face-to-face interviewing questionnaires and analyzed using SPSS version 21.0. Binary logistic regression analyses were performed to identify predictors.

**Results:**

The prevalence of chronic kidney disease diagnosed by Cockcroft-Gault equation and Modification of Diet in Renal Disease equation was 14.6% and 7.7% respectively. This finding shows the prevalence of chronic kidney disease among Diabetic patients was low. Age 50–59 years [(AOR= 4.0; 95% CI:(1.2, 13)] by Cockroft-Gault equation (CG), age 60–69 years [(AOR=5.8 95% CI:(1.5,21.0)] by Modification of Diet in Renal Disease (MDRD) and (AOR;22.9 95%CI:7.1,74.2) by CG, age 70 years and above (AOR=4.7; 95 CI: 1.1, 19.7) by MDRD and (AOR= 22.9; 95%CI:7.1,74.2) by CG, BMI (AOR=0.2; 95% CI:0.1,0.4) by CG, and previous kidney disease (AOR=6.2 95%CI:2.0,8.4) by MDRD and (AOR;4.6 95%CI:1.9,10.8) C-G equation have a significant association with chronic kidney disease after an adjustment done.

**Conclusion:**

The prevalence of chronic kidney disease among Diabetic patients in this study was lower. Age, BMI, and previous recurrent kidney disease were associated with chronic kidney disease. Preventive measures like giving health education and screening of patients with risk factors should get more attention.

## Introduction

Chronic kidney Disorder (CKD), a progressive disease, abnormalities of kidney structure and function for greater than 3 months with an estimated glomerular filtration rate (eGFR) less than 60 mL/min/1.73 m2 and/ or evidence of kidney damage, including persistent albuminuria - defined as greater than 30 mg of urine albumin per gram of urine creatinine (1). It has five stages which are determined by glomerular filtration rate, the process by which the kidney filters the blood, removing excess wastes and fluids (2). Even though there are many causes of CKD, its two most common causes which are responsible for two-thirds of all cases are diabetes (44%) and high blood pressure/hypertension (29%) (3). Studies revealed that Diabetic nephropathy affects approximately 20–40 % of individuals who have diabetes, making it one of the most common complications related to diabetes (4,5).

CKD is a global public health problem with its prevalence estimated to be between 8% and 16%. Despite various measures, the burden of renal disease is likely to escalate since both population age and prevalence of diabetes are projected to increase dramatically (6,7). Furthermore, the rapid increase in the prevalence of risk factors, such as hypertension, and obesity, has increased the burden of CKD, making CKD an important socioeconomic and public health problem. Moreover, a study revealed that some socioeconomic factors, history of kidney stones, and the use of nephrotoxic medications are its contributing factors (8). The prevalence of the chronic disease is increasing in Ethiopia mainly due to urbanization, sedentary lifestyles, and population growth. There have been only a few studies that examined CKD prevalence among diabetic patients in Ethiopia (9,10). Besides, renal complications of diabetes may go unrecognized due to limited diagnostic resources. Since there is no renal registry that is established to document the epidemiology of the renal disease, the prevalence of CKD among adult diabetics in Ethiopia has not been well described. Therefore, this study was designed to assess the prevalence of CKD and associated factors among adults attending the Police Hospital diabetic Clinic in Addis Ababa.

## Methods

**Study design and setting**: Hospital-based cross-sectional study design was conducted in a federal police hospital, Addis Ababa, from October to December 2017. Federal police hospital is one of the government hospitals in Addis Ababa, Ethiopia, which was established in 1962, under the Ethiopian Federal Ministry of Health. It provides various health services including DM treatment to the police officers and their families. During the study, there were about 1800 diabetic patients enrolled for follow-up care and the patients visited the clinic for follow-up care every 3 months. All systematically selected diabetic patients attending the hospital outpatient diabetic clinic for follow-up during the data collection period were the study population. Diabetes patients greater than 18 years old who were attending the clinic and registered at chronic care were eligible for the study. However, pregnant women were excluded from the study to avoid the effect of gestational diabetes. The sample size was calculated using single population proportion formula by considering the following parameters: 10.4% proportion of chronic Kidney Disease from a previous study (10), 95% confidence interval, and 5% margin of error. Since the total number of patients on follow-up care was less than 10,000, the sample size was adjusted by using the finite population correction formula. Then, by considering the non-response rate of 10%, the total sample size calculated was 363. Every 4^th^ diabetic patient was selected using a systematic sampling technique and the first subject was determined by a simple random sampling method from the first 4 patients.

**Data collection and measurement**: Data were collected using a structured questionnaire adapted from related literature (4, 12, 14,15, 16, 17,18, 19), and then it was translated to Amharic (the local language) for data collection after checking its consistency. The questionnaire was pretested on 5% of similar patients in Zewditu Memorial Hospital in Addis Ababa. The questionnaire contains socio-demographic status, personal and family health history, and lifestyle behavioral factors. History of taking medications with nephrotoxic potential was recorded, Weight and height were measured, and BMI was calculated by the data collectors. Similarly, laboratory results of blood tests, which were done every 3 months, were filled from records in the Hospital. To assure the quality of data, data collectors and supervisors were trained for one day.

**Measurement**: For this research, CKD was defined as eGFR less than 60ml/min/1.73m^2^ by both equations (MDRD and C-G). High blood glucose level and Serum cholesterol level were defined as those who has FBS>150mg/dl and cholesterol level>200mg/dl during the study period respectively. Physical exercise was defined as having physical activities greater than 150 min per week. Alcohol consumption was also measured as patients who consumed greater than two alcoholic beverages per day. Former alcohol consumer was defined as those who consume greater than two alcoholic beverages but has not consumed for the last 12 months before the study and current smoker was defined as those who has smoked greater than 100 cigarettes in their lifetime and has smoked in the last 28 days before the study. Ex-smoker was defined as those who have smoked greater than 100 cigarettes in their lifetime but have not smoked in the last 28days before the study. Knowledge of CKD was defined as respondents' understanding of kidney disease symptoms, treatment, management, and risk factors, and those who answered more than 50% of the question were considered as knowledgeable.

**Data analysis**: A STROBE checklist was used to analyze and report data (11). Data were entered into EpiData version 3.1 and then exported to SPSS version 21 for analysis. Descriptive analyses were done. Bivariate logistic regression analysis was done to select candidate variables for multivariate logistic regression analysis. Variables with a p-value less than 0.25 in the bivariate analysis were considered as a candidate to be entered into the final model. Odds ratio (OR) with 95% confidence interval (CI) were calculated to see the predictor variables and p-value < 0.05 was considered statistically significant.

**Ethical approval**: Ethical clearance was obtained from the ethical review board of the institute of health, Jimma university, from the Addis Ababa Health Bureau ethical committee, and from the Ethical Committee at Federal Police Commission Referral Hospital. Each participant was informed about the objectives and benefits of the research and its findings, preceding the data collection. Verbal consent was obtained from each participant prior to enrollment. Names were not used in collecting the data from the medical files, and confidentiality were maintained by keeping the data collection forms locked in a secure cabinet.

## Results

**Description of the study participants**: A total of 362 diabetic patients participated in our study making the overall response rate 99.7%. The mean (SD) of the age of study participants was 55.4 (±13.63) years. Fifty-eight percent of the study participants were in the age group of less than 60 years and about 69% were males. More than three fourth (81.2%) of study participants were married. A majority (42.5%) of the study had primary education and more than three fourth (85.4%) had more than 500 Ethiopian birr monthly income (Table 1).

**Table 1:** Sociodemographic characteristics of study participants in a federal police hospital, Addis Ababa, 2017

Variables		Frequency	Percent
**Age (year)**	18–49	119	32.9
	50–59	91	25.1
	60–69	96	26.5
	≥70	56	15.5
**Sex**	Male	251	69.3
	Female	111	30.7
**Educational status**	Illiterate/primary	89	24.6
	Secondary	154	42.5
	Higher	119	32.9
**Marital status**	Unmarried	15	4.1
	Married	294	81.2
	Divorced/Widowed	53	14.6
**Residence**	Urban	296	81.8
	Rural	66	18.2
**Monthly income**	≤ 500	53	14.6
**(Ethiopian Birr)**	>500	309	85.4

**Prevalence of Chronic Kidney Disease**: The prevalence of CKD diagnosed by the C-G equation was 14.6% (n=53) and the prevalence diagnosed by the MDRD equation was 7.7% (n=28). Using the MDRD equation the stages of CKD were diagnosed as; stage 3 were 6.9% (n=25), stage 4 were three individuals, and none on stage 5. Whereas, as per the CG equation, among the 53 individuals, 13.5% (n=49) were on stage 3, four individuals were on stage 4 and none of them were on stage 5. This indicates the prevalence of CKD is higher at stage 3 and none at stage 5.

**Factors associated with chronic kidney disease**: At bivariate analysis age, sex, income, BMI, DM duration, types of medication, history of hypertension, duration of hypertension, cardiovascular disease, previous history of kidney disease, cigarette smoking, alcohol intake, and knowledge were predictors. A multivariable logistic regression analysis; age, BMI, and previous kidney disease were found to be predictors. Attendants whose age was between 50–59 years were 4 times more likely (AOR=4.0; 95% CI (1.2,13) to develop chronic kidney disease compared to patients whose age was under 50 years by CG equation. Similarly, attendants whose age was between 60–69 years by MDRD (AOR=5.8; 95% CI:1.5, 21.0) and by CG (AOR=8.1; 95% CI (2.6, 25.0) were about 6 and 8 times more likely to develop chronic kidney disease respectively when compared to patients whose age was under 60 years. Respondents whose age was 70 years and beyond by MDRD (AOR=4.7; 95% CI: (1.1,19.7) and by CG (AOR=22.9; 95% CI (7.1,74.2) were about 5 and 23 times more likely to develop chronic kidney disease respectively when compared to patients whose age was under 60 years. Patients with high BMI were significantly associated with chronic kidney disease when using the C-G equation. Obese patients were 80% less likely to develop chronic kidney disease than those who were not obese (AOR=0.2; 95% CI: 0.1, 0.4). Those who have been recurrently diagnosed to have any form of kidney disease were 6 and 5 times more likely to develop chronic kidney disease when using MDRD (AOR;6.2 95% CI:2.0,8.4) and C-G equation (AOR;4.6 95%:1.9,10.8) respectively (Table 2).

**Table 2:** Bivariate and Multivariate analysis of factors associated with CKD among participants in Federal police hospital Addis Ababa, 2017

Variable	MDRD			C-G		
	COR	AOR	P-value	COR	AOR	P-value
**Age**						
18–49	1			1	1	
50–59	2.7(0.6,11.2)	3.2(0.8,13.9)	0.107	0.1(0.0,0.2)	4.0(1.2,13)	0.028
60–69	5.5(1.5,20.1)	5.8(1.5,21.0)	0.009	0.2(0.1,0.4)	8.1(2.6,25.0)	<0.001
≥70	5.5(1.5,22.2)	4.7(1.1,19.7)	0.033	0.3(0.2,0.7)	22.9(7.1,74.2)	<0.001
**BMI**						
Non-obese	1			1	1	
Obese	0.7(0.3,1.9)			0.2(0.9,0.6)	0.2(0.1,0.4)	<0.001
**Previous kidney** **disease**						
Yes	6.3(2.7,14.7)	6.2(2.6,15.1)	<0.001	4.1(2.0,8.4)	4.6(1.9,10.8)	<0.001
No	1			1	1	

## Discussion

This study found the prevalence of CKD to be 7.7% and 14.6% by using MDRD and C-G equations respectively. Previous studies conducted in Addis Ababa and southern Ethiopia have shown that CKD prevalence is between 10.4% by MDRD and 19.1% by C-G equation and 18.2% by MDRD and 23.8% by C-G equation respectively. The estimated prevalence of CKD using both equations in the current study is lower than previously conducted studies in Ethiopia (9, 10). On the contrary, a study done in Bangladesh and Mediterranean area using the MDRD equation, 13.1%, and 13.4% respectively, were higher than the estimated prevalence of CKD obtained from this study using the same equation (14, 15). The estimated prevalence of CKD using C-G equation was higher than the estimated prevalence of CKD reported in sub-Saharan Africa 12.4% and lower than the estimated prevalence of CKD in Tanzania 24.7% (13, 17). The possible reason for the discrepancy is the estimated prevalence of CKD might be due to the unavailability of the patients during the data collecting period. Patients with end-stage renal disease (stage 5 CKD) referred to other hospitals for possible dialysis were unavailable at the time of the survey. On the other hand, the difference might be due to the difference in population, as this study was in a police Hospital, somehow different from the general population.

Advanced age groups were found to have a significant association with chronic kidney disease using both MDRD and C-G equations. The finding is consistent with other studies conducted in China and southern Ethiopia (9, 16). In various studies conducted worldwide, older age was found to be the strongest predictor of lower estimated GFR and it has been significantly associated with increased incidence of CKD among diabetic patients. Since age increases the risk, long-term reduction of CKD morbidity and mortality requires more attention to early detection and prevention of CKD using various screening tools among the older diabetic population.

Being obese was inversely associated with the disease using the C-G equation. This is contrary to the study done in India (20). The difference might be due to the difference in population and study setting, as this study was in a police Hospital, somehow different from the report from India which was a community-based study.

Previous recurrent attack of kidney disease was also significantly associated with CKD irrespective of the equation were used. Those who had been diagnosed with a kind of kidney disease previously were found to be more likely to develop current chronic kidney disease compared to those who had never been diagnosed to have kidney disease using both equations. A study done in Tanzania showed an association when the analysis was done using bivariate but was not associated when entered into multivariate logistic regression (17). Even though variable hypertension shows an association with CKD in other studies, it is not statistically significant in our study.

The prevalence of chronic kidney disease in diabetic patients using both equations is high. Age of the patients, having a history of previous kidney disease and BMI were associated with chronic kidney disease. Therefore, alleviating modifiable and preventable factors that lead to kidney disease is recommended. Further, the provision of persistent health education and training for those who are diagnosed to have diabetes mellitus reduces the magnitude of the problem. The study has the following limitations. It was conducted in only one hospital which may affect the generalization of the finding and since the study was a cross-sectional study, the possibility of recall bias may result in underreporting of the results.
